# Low skeletal muscle area is a risk factor for mortality in mechanically ventilated critically ill patients

**DOI:** 10.1186/cc13189

**Published:** 2014-01-13

**Authors:** Peter JM Weijs, Wilhelmus GPM Looijaard, Ingeborg M Dekker, Sandra N Stapel, Armand R Girbes, Heleen M Oudemans-van Straaten, Albertus Beishuizen

**Affiliations:** 1 Department of Nutrition and Dietetics, Internal Medicine, VU University Medical Center Amsterdam, De Boelelaan 1117, Amsterdam, The Netherlands; 2 Department of Intensive Care Medicine, VU University Medical Center Amsterdam, De Boelelaan 1117, Amsterdam, The Netherlands; 3 Department of Nutrition and Dietetics, Amsterdam University of Applied Sciences, Dr. Meurerlaan 8, Amsterdam, The Netherlands; 4 Department of Intensive Care Medicine, Medisch Spectrum Twente, Ariënsplein 1, Enschede, The Netherlands; 5 Institute for Cardiovascular Research, VU University Medical Center Amsterdam, De Boelelaan 1117, Amsterdam, The Netherlands

## Abstract

**Introduction:**

Higher body mass index (BMI) is associated with lower mortality in mechanically ventilated critically ill patients. However, it is yet unclear which body component is responsible for this relationship.

**Methods:**

This retrospective analysis in 240 mechanically ventilated critically ill patients included adult patients in whom a computed tomography (CT) scan of the abdomen was made on clinical indication between 1 day before and 4 days after admission to the intensive care unit. CT scans were analyzed at the L3 level for skeletal muscle area, expressed as square centimeters. Cutoff values were defined by receiver operating characteristic (ROC) curve analysis: 110 cm^2^ for females and 170 cm^2^ for males. Backward stepwise regression analysis was used to evaluate low-muscle area in relation to hospital mortality, with low-muscle area, sex, BMI, Acute Physiologic and Chronic Health Evaluation (APACHE) II score, and diagnosis category as independent variables.

**Results:**

This study included 240 patients, 94 female and 146 male patients. Mean age was 57 years; mean BMI, 25.6 kg/m^2^. Muscle area for females was significantly lower than that for males (102 ± 23 cm^2^ versus 158 ± 33 cm^2^; *P* < 0.001). Low-muscle area was observed in 63% of patients for both females and males. Mortality was 29%, significantly higher in females than in males (37% versus 23%; *P* = 0.028). Low-muscle area was associated with higher mortality compared with normal-muscle area in females (47.5% versus 20%; *P* = 0.008) and in males (32.3% versus 7.5%; *P* < 0.001). Independent predictive factors for mortality were low-muscle area, sex, and APACHE II score, whereas BMI and admission diagnosis were not. Odds ratio for low-muscle area was 4.3 (95% confidence interval, 2.0 to 9.0, *P* < 0.001). When applying sex-specific cutoffs to all patients, muscle mass appeared as primary predictor, not sex.

**Conclusions:**

Low skeletal muscle area, as assessed by CT scan during the early stage of critical illness, is a risk factor for mortality in mechanically ventilated critically ill patients, independent of sex and APACHE II score. Further analysis suggests muscle mass as primary predictor, not sex. BMI is not an independent predictor of mortality when muscle area is accounted for.

## Introduction

Patients admitted to the intensive care unit (ICU) are often severely ill, and many have muscle (protein) catabolism, muscle weakness, and/or atrophy, all linked to an increased morbidity and mortality [1]. Patients with inadequate energy and protein intake are at risk for complications, including infections, acute respiratory distress syndrome, need for surgery, and renal failure [2,3]. Adequate reserves of body protein and fat mass at admission to the ICU may be crucial to recovery and survival.

A number of meta-analyses [4,5] have been published on the observation of a lower hospital mortality in critically ill patients with a body mass index (BMI) in the overweight (BMI, 25 to 30) and obese (30 to 40) range [6]. Additionally, more recent studies [7-9] have reestablished these findings. This so-called obesity paradox is also observed in chronic conditions such as hemodialysis. Increasing evidence points to preserved muscle protein as part of the explanation [10].

Although BMI can easily be assessed in very large cohorts, only limited observations are available for actual and reliable measurement of protein mass. Recently, Baracos *et al*. developed a technique for muscle mass assessment by analyzing computed tomography (CT) scans in cancer patients [11,12]. They showed that sarcopenic obese patients, defined by a high BMI and a low muscle mass, have a higher mortality [13,14]. This observation suggests that “fat is good, but muscle is better.” High BMI is not only associated with an increased fat mass, but also with an increased fat-free mass [15]. At the same time, a normal BMI is not exclusively based on a normal fat mass, but may be the result of a high fat mass with a relatively low muscle mass. Assessment of muscle mass might help to optimize protein and energy balance and improve risk assessment in the ICU [16].

Therefore, we investigated the relation between muscle mass as assessed from CT scans, as well as BMI, and outcome in a cohort of mechanically ventilated critically ill patients.

## Materials and methods

### Patients

This is a retrospective analysis in a mixed medical-surgical group of patients admitted to the ICU of a university hospital in the period 12/2003 to 09/2012. Inclusion criteria were age of 18 years or older, ICU stay of at least 4 days, mechanical ventilation during ICU stay, and an “early” CT scan of the abdomen (including L3 slice) made within a time frame of 1 day before admission to 4 days after admission for diagnostic and/or interventional reasons. Exclusion criteria were CT scans not meeting quality checks (artifacts and muscle outside scanned frame) and missing data on body weight and height.

Patients eligible for this study were identified by searching the patient data-management system (PDMS) for patients who fulfilled the inclusion criteria and had the words “CT” and/or “scan” (excluding “bladder scan”) in the nursing-notes section.

Demographic and clinical data including sex, age, weight, height, BMI, Acute Physiologic and Chronic Health Evaluation (APACHE) II score, admission diagnosis, length of stay (LOS) in the ICU and hospital, as well as ICU-, 28-day-, and hospital mortality were obtained from the PDMS (Metavision; IMDsoft, Tel-Aviv, Israel) and hospital information system (Mirador; iSOFT Nederland BV, Leiden, The Netherlands). The study was approved by the Institutional Review Board of the VU University Medical Center. The need for informed consent was waived because of the retrospective nature of the study using coded data obtained from routine care.

### CT scan analysis

CT scans were imported from the local radiology system and assessed for quality. First, the third lumbar vertebra (L3) was identified on abdomen CT scans. This landmark was chosen because of its established correlation with whole-body muscle mass [17-19]. The L3 slice provides information on a number of muscles: the erector spinae-, quadratus lumborum-, psoas-, transversus abdominis-, interior- and exterior oblique-, and rectus abdominis muscles. The L3 slice was isolated and stored for later analysis.

CT scan analysis was performed by using Slice-O-matic version 4.3 and 5.0 (TomoVision, Montreal, QC, Canada) by trained personnel. Muscle tissue was identified by using boundaries in Hounsfield Units set to -29 to +150 [18]. The software computed a muscle surface area in cm^2^ by multiplying the pixel area by the amount of pixels identified as muscle.

BMI categories were used as defined by the World Health Organization [6].

### Statistics

Fisher Exact- and χ^2^ tests were used to compare categoric variables and Mann–Whitney *U* and *T* tests for continuous variables not normally distributed, and *t* test for continuous variables with a normal distribution.

ROC curve analysis was used to define muscle-area cutoff values best fit to predict hospital mortality in female and male patients separately.

Backward stepwise (Wald) regression analyses were performed, with hospital mortality as outcome variable. Independent variables were low muscle area (below sex-specific cutoff, y/n), sex, BMI, APACHE II score, and admission diagnosis. Additionally, Kaplan-Meier curve analysis was performed to show the relation between the different muscle-area groups and mortality.

We also investigated muscle area as a continuous variable (expressed per 10 cm^2^) with stepwise logistic analysis. Last, another model was investigated by using the cutoff value for females for males as well, additional to the cutoff values for males. In this way, high-, medium-, and low-muscle area groups were created. SPSS 20 (SPSS Inc., Chicago, IL, USA) was used for statistical analysis. A *P* < 0.05 was considered statistically significant.

## Results

**Figure 1:**
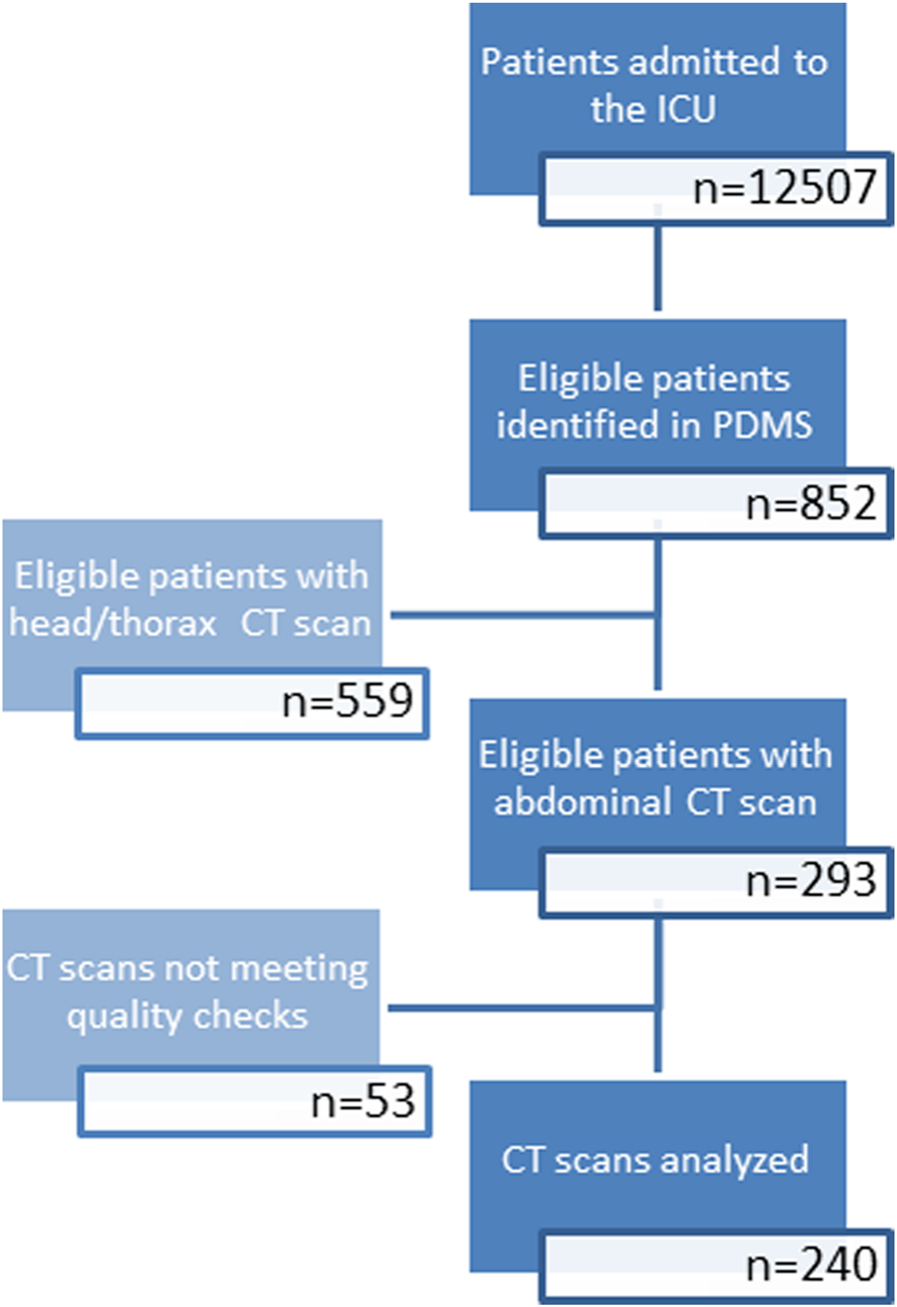
Consort diagram showing the inclusion process.

Figure 1 shows the consort diagram.

During the study period, 12,507 patients were admitted to the ICU with a mean APACHE II score of 17.44 (SD, 8.45). Of these, 852 (6.8%) patients fulfilled the inclusion criteria, and CT scans were retrieved of 293 patients. Quality check of the CT scans revealed that 19 scans did not display all required muscles, 24 scans contained artifacts, and in 10 cases, the quality of the scan was considered insufficient for analysis. Therefore, 53 scans were excluded, and scans of 240 patients remained for further analysis. The study population therefore represents 35.5% of the patients fulfilling the inclusion criteria (ventilated patients with an ICU stay of at least 4 days) and 82% of the patients with an early CT scan. Mean APACHE II score of the study population was 23.4 (SD, 8.1). The mean interval between ICU admission and the CT scan was 0.74 (SD, 1.18) days.

**Figure 2:**
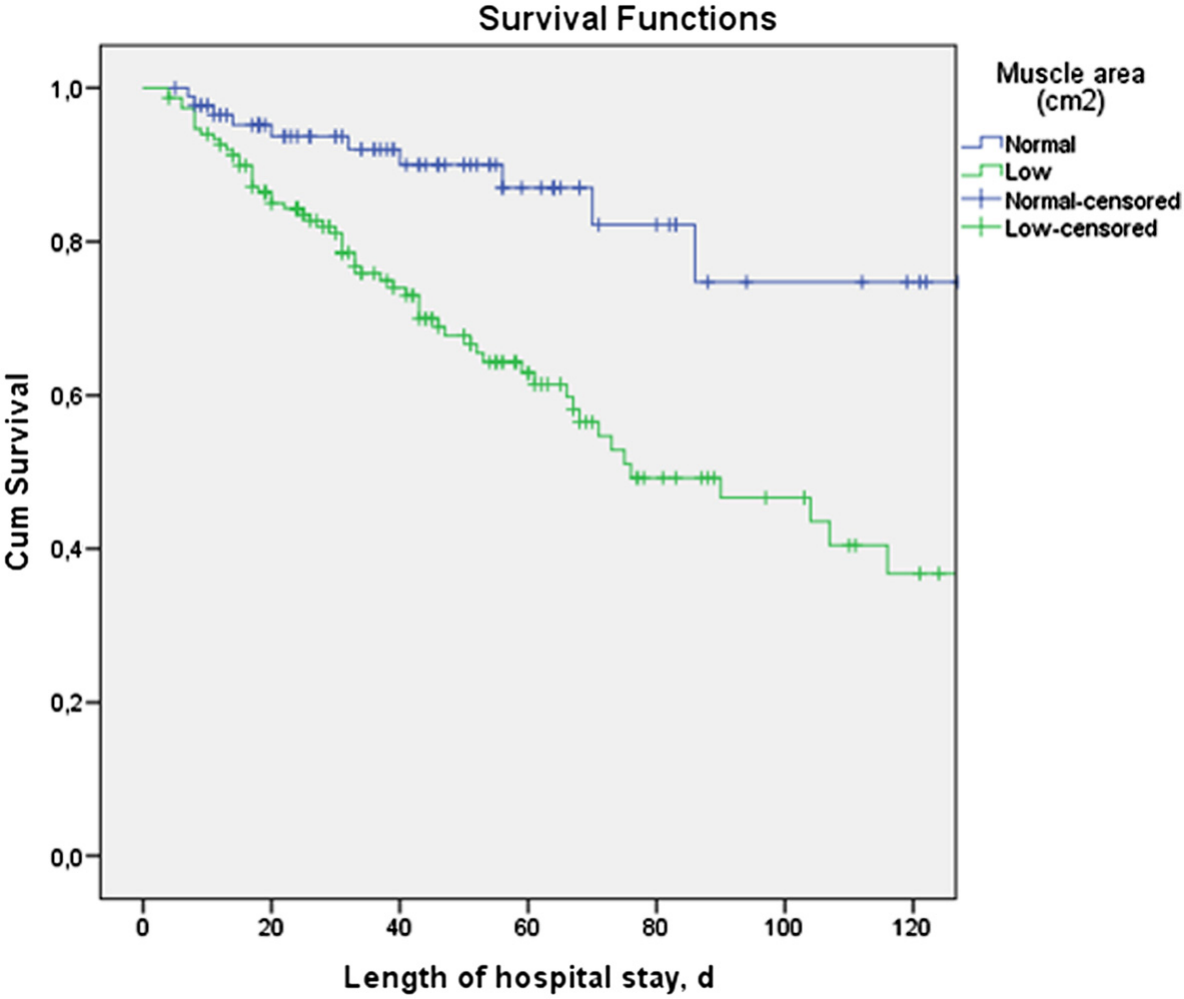
**Kaplan-Meier survival plot for low- and normal-muscle area group (log rank test, *P <*** **0.001).**

Baseline characteristics of all patients, and of those with low and normal muscle area separately, are presented in Table 1; 240 patients were included (94 F, 146 M). Mean muscle area for females was lower than for males (102 ± 23 cm^2^ versus 158 ± 33 cm^2^; *P* < 0.001). We therefore determined cutoff values for low-muscle area for males and females separately. ROC curve analysis provided cutoff values of 110 cm^2^ for females and 170 cm^2^ for males. A low muscle area was observed in 63% of patients for both females (below 110 cm^2^) and males (below 170 cm^2^). Mortality was 29% and higher in females than in males (37% versus 23%; *P* = 0.028). Patients with low muscle area had a higher hospital mortality (38.2% versus 12.5%; *P* < 0.001), in females (47.5% versus 20.0%, *P* = 0.008) as well as in males (32.3% versus 7.5%; *P* < 0.001). Figure 2 shows the difference in mortality as Kaplan-Meier curves for the combined (females and males) low-muscle area group versus normal muscle-area group.

**Table 1:** Patient characteristics and outcome for normal- and low-muscle area patient group

	**Normal muscle-area patients *n*** **= 88**		**Low muscle-area patients *n*** **= 152**		**All patients *N*** **= 240**		**Fisher exact test or student *t *-test, -value**
	**Mean**	**SD**	**Mean**	**SD**	**Mean**	**SD**	
Female gender, %	40		39		39		0.892
Age, y	52.3	19.0	63.6	15.7	59.5	17.8	**<0.001**
APACHE II score	21.7	8.6	24.4	7.7	23.4	8.1	**0.013**
Weight, kg	80.2	15.6	73.6	15.1	76.0	15.6	**0.001**
Height, cm	174.4	9.7	173.0	9.5	173.5	9.5	0.281
BMI, kg/m^2^	26.3	4.3	24.4	4.1	25.1	4.2	**0.001**
Muscle mass, kg	22.0	4.2	16.6	3.6	18.6	4.6	**<0.001**
Muscle index, cm^2^/m^2^	54.5	8.7	39.4	8.3	45.0	4.6	**<0.001**
Muscle area, cm^2^	167.1	35.8	119.1	30.2	136.7	39.8	**<0.001**
Admission diagnosis, %							
Cardiovascular	5.7		14.5		11.2		**0.009**
Metabolic/Renal	3.4		1.3		2.1		**(χ**^**2 **^**test)**
Neurologic	5.7		3.9		4.6		
Postresuscitation	3.4		2.0		2.5		
Postsurgery	26.1		34.2		31.2		
Respiratory insufficiency	9.1		15.8		13.3		
Sepsis	4.5		7.2		6.2		
Trauma	35.2		14.5		22.1		
Others	6.8		6.6		6.7		
Length of ventilation, d (median; IQR)	15	8-28	13	8-23	14	8-25	0.387
ICU length of stay, d (median; IQR)	17	9-30	16	10-28	16	9-28	0.768
Hospital length of stay, d (median; IQR)	42	20-64	41	24-66	41	22-65	0.930
ICU mortality, %	10.2		23.7		18.8		**0.010**
28-d mortality, %	5.7		21.7		15.8		**0.001**
Hospital mortality, %	12.5		38.2		28.8		**<0.001**
Survivor analysis, *n*	77		93		170		
To nursing home, %	11.7		24.7		18.8		**0.032**

APACHE, Acute Physiologic and Chronic Health Evaluation; BMI, Body mass index; IQR, interquartile range.

Stepwise regression analysis showed that muscle area, sex, and APACHE II score were independent predictors for hospital mortality, whereas BMI and admission diagnosis were not. Odds ratio for low-muscle area was 4.3 (95% CI, 2.0 to 9.0; *P* < 0.001). Table 2 shows results for three models, first the model with muscle area as a continuous variable, second for sex-specific cutoff values, and third for sex-combined cutoff values. In the sex-combined approach, creating low-medium-high muscle area groups, the low-muscle area group (<110 cm^2^) contains 63% of females and 8% of males, with similar hospital mortality rate (47.5% for females, 45.5% for males).

**Table 2:** Backward stepwise (Wald) regression analyses

	**Muscle area (continuous) (per 10 cm**^**2**^**)**			**Low-muscle area versus normal**			**Low and medium muscle area versus high**		
				**Low < 110 cm**^**2 **^**for females**			**Low <110 cm**^**2**^		
				**Low < 170 cm**^**2 **^**for males**			**Medium 110 to 170 cm**^**2**^		
							**High >170 cm**^**2**^		
**Step 1**	**OR**	**95% CI**	***P *** **value**	**OR**	**95% CI**	***P *** **value**	**OR**	**95% CI**	***P *** **value**
Muscle area	0.82	.73 – .93	**.001**	3.86	1.80 – 8.26	**.001**	2.93	1.58 – 5.42	**.001**
Sex	1.50	.67 – 3.34	.320	.54	.29 – 1.01	.053	1.43	.66 – 3.09	.365
BMI	0.96	.89 – 1.03	.268	.95	.88 – 1.03	.222	.96	.89 – 1.03	.238
APACHE II score	1.06	1.03 – 1.11	**.001**	1.07	1.03 – 1.11	**.001**	1.07	1.03 – 1.11	**.001**
Admission diagnosis	0.96	.85 – 1.10	.577	.97	.85 – 1.11	.666	.97	.85 – 1.10	.625
Constant	3.01		.341	.16		.118	.02		.012
**Step 3 or 4**									
Muscle area	0.84	.77 – .92	**<.001**	4.27	2.03 – 9.00	**<.001**	2.71	1.69 – 4.34	**<.001**
Sex				.50	.27 – .92	**.025**			
APACHE II score	1.06	1.02 – 1.1 7	**.002**	1.07	1.03 – 1.11	**.001**	1.07	1.03 – 1.11	**.001**
Constant	0.83		.812	.04		<0.001	.01		<.001

## Discussion

This study shows that low skeletal muscle mass is an independent risk factor for mortality in mechanically ventilated critically ill patients. This observation is independent of sex and APACHE II score. The study also shows that when muscle area is accounted for, BMI appears to have no impact on mortality. The study group represents a more severely ill population still present in the ICU at day 4. Skeletal muscle mass was obtained from CT scans made for clinical reasons during the first days of ICU admission and expressed as skeletal muscle area at the level of the third lumbar vertebra.

Our findings are in line with a recent study by Moisey *et al*. [20] in 149 injured elderly ICU patients. They also found that low muscle mass, as assessed by CT scans, was associated with mortality. Our observations are made in an ICU representative age group, and not in an elderly population, as in Moisey’s population. This study further adds new ICU-specific cutoff values. When the cutoff value for females was also applied for males (right panel in Table 2), a very high-risk group became apparent, of which one of two patients died during hospital stay. Reason for the higher mortality in our study with respect to age is that our study group represents a more severely ill population still present in the ICU at day 4, and Moisey took CT scans on admission [20].

Several studies have shown a relation between high BMI and lower mortality in ICU patients [4,5,7,8], but results are not consistent [21]. However, a recent very large observational cohort study in 154.308 patients provided more evidence on this so-called obesity paradox [9]. The study found that overweight and obese patients with BMI up to 40 had the lowest risk of death. The authors argued that fat mass may play an important role in the immunologic response of the patient under stressed conditions in the ICU. However, our study shows that muscle area is a stronger predictor than BMI. BMI is based on weight and height; however, weight includes both fat mass and muscle mass. For healthy obese females, we have shown that the mean value for fat-free mass increases from 40 to almost 60 kg between BMI 25 and BMI 50 [15]. Because heavier people carry a significant weight, they are likely to have an increased muscle mass along with an even much more increased fat mass. As a result, the assessment of BMI, without any insight into body composition, does not prove that increased fat mass is beneficial for ICU patients. A significant problem may also be the limited accuracy of weight assessment in ICU patients. In a large group of almost 1,500 oncology patients, Martin et al. [22] showed that the poor prognosis based on weight loss, low muscle index, and muscle attenuation was independent of weight status (underweight, normal weight, overweight, and obesity).

In addition, it was shown before that sarcopenic obesity, being the combination of a high BMI and a low muscle mass, was related to a poor prognosis, in particular in cancer patients [12]. The study by Martin *et al*. extends the finding that low muscle index is prognostic for mortality, independent of weight status and not only for obese patients. Moisey *et al*. added the risk of low muscle for mortality in elderly injured ICU patients, while not showing a relation between fat tissue and mortality [20].

Previously we showed that reaching both protein and energy targets in critically ill patients had a positive impact on survival [23,24]. In an earlier analysis, we were able to show beneficial effects of optimal nutrition only in females [23]. Females by nature have a lower muscle mass and thus a lower body-protein mass as compared with males. We therefore hypothesized that the lower protein mass in females might be prone to reaching a critical point earlier under the stressed conditions of ICU admission. Our present study seems to confirm this hypothesis, because we found that females had a lower muscle mass than males. In addition, if muscle area was introduced as a continuous variable and not as a sex-related cut-off category, sex disappeared as independent predictor of mortality.

When we applied both sex-specific cutoffs to both females and males, this high-medium-low group in the backward stepwise regression analysis obviated sex as a predictor. Therefore, we stress that the muscle mass and mortality issue is primarily a mass effect and that the gender issue is considered secondary. The gender/muscle mass relation also sheds new light on previous studies reporting that female patients have a higher mortality when developing nosocomial infections in the ICU [25] or after cardiac surgery [26]. Reduced muscle mass in females may explain these observations and may prove to be an important denominator of poorer outcome.

The present study should be considered as a proof-of-concept study. CT scans are an important tool to assess nutritional status of cancer patients, because scans are part of their routine monitoring. However, CT scans are not routinely performed in ICU patients and cannot be used as such because of X-ray exposure, lack of time, expertise, and high costs. Using CT scans for longitudinal monitoring is unfeasible, and for the intensive care population, alternatives for the CT scan should be explored. Caporossi *et al*. [27] assessed muscle mass by measuring thickness of the adductor pollicis muscle. Low thickness of this thumb muscle was associated with a 6 times higher risk of death in 246 ICU patients. In addition, low quadriceps femoris muscle mass, as assessed by ultrasound, was associated with longer ICU stay [1,16]. An advantage of using these approaches is that multiple longitudinal measures are easily obtained, and therefore allow long-term monitoring of muscle mass in the ICU patient [28,29]. An alternative option could be to monitor fat-free mass or phase angle by using bioelectrical impedance [30].

If the independent relation between low muscle mass and higher mortality is causative, the question arises whether therapeutic interventions can positively influence this relation. Possible interventions include optimization of nutrition, muscle training, or electrical muscle stimulation. The dosing of the nutritional protein component remains controversial. Most nutritional guidelines express protein needs per kilogram body weight, whereas only Ishibashi *et al.*[31] were able to advise 1.5-gram protein per kg fat-free mass. Low muscle mass is a likely indicator of undernutrition and/or poor mobility. If muscle mass could be assessed in daily clinical practice, this information could influence daily treatment [32]. Controversy exists about the timing of starting feeding in ICU patients. Early feeding could suppress autophagy, and protein seems the most important factor [33,34]. Monitoring muscle mass may at least contribute to the understanding of protein needs for ICU patients and provide a means to evaluate the effect and timing of different nutrition regimens.

The present study has strengths and limitations. It is the first study proving that measured muscle area by using novel techniques in critically ill patients is a predictor of patient outcome in a general ICU population, confirming the relation found in cancer patients and elderly injured patients. It also shows that muscle mass is a stronger predictor for outcome than BMI, suggesting that the patient’s muscle reserve is more important than his fat reserve. Limitations include that it is unknown whether CT scan-derived muscle mass truly relates to total muscle mass in ICU patients. However, for healthy subjects [19] and cancer patients [13], a good relation has been shown. In addition, the timing of CT scanning was not standardized because scans performed for clinical reasons were used; this may have introduced some selection bias. Patients in whom a CT scan is made may not be representative for all ICU patients, which could be another source of selection bias. However, all scans were made within the first 4 days of ICU admission. Finally, CT scanning is not a feasible tool for routine monitoring of muscle mass, and alternative imaging tools such as ultrasound or bio-impedance should be explored.

## Conclusion

Low skeletal muscle area is a risk factor for mortality in our cohort of mechanically ventilated critically ill patients who had a CT scan made during the first days of ICU admission. This observation was independent of sex and APACHE II score. Additional analysis suggested muscle mass as a primary predictor, not gender. Furthermore, when muscle area was accounted for, BMI was not a predictor of mortality. The ICU-specific cut-off values as found in our study need external validation.

## Key messages

• Low skeletal muscle area is an independent risk factor for mortality in mechanically ventilated critically ill patients in an ICU representative age group.

• The effect of muscle is independent of sex and APACHE II score.

• When muscle area is accounted for, BMI appears to have no impact on mortality.

## Abbreviations

APACHE: Acute Physiologic and Chronic Health Evaluation; BMI: body mass index; CT: computed tomography; ICU: intensive care unit; L3: third lumbar vertebra; LOS: length of stay; OR: odds ratio; PDMS: patient data-management system.

## Competing interests

The authors declare that they have no competing interests.

## Authors’ contributions

PW, AB, WL, HO, and AG designed the study. WL, AB, and SS obtained the data. WL and ID analyzed the CT scans. PW performed statistical analysis. All authors contributed in drafting the manuscript and authorized the final manuscript.
